# Causal Effect of Education on Tobacco Use in Low-and-Middle-Income Countries

**DOI:** 10.1093/ntr/ntad056

**Published:** 2023-04-05

**Authors:** Mustafa Utku Özmen

**Affiliations:** Department of Economics, TOBB University of Economics and Technology, Ankara, Turkey

## Abstract

**Introduction:**

The prevalence of smoking is unequally distributed across certain groups. One significant dimension is education inequality, where higher smoking prevalence is generally observed in lower-educated groups. However, studies investigating educational inequality are mostly associative. Meanwhile, studies carrying out a causal investigation focus typically on developed countries. In this study, we consider a panel of low-and-middle-income countries (LMICs) to investigate the causal link between education and smoking behavior.

**Aims and Methods:**

We use detailed micro-level household surveys for 12 LMICs where the duration of compulsory schooling has been extended. By identifying the individuals subject to higher compulsory schooling and using the exogenous variation in education caused by the increase in the duration of compulsory schooling, we estimate the causal impact of education on tobacco consumption. We rely on regression analysis to estimate the effect.

**Results:**

Our results reveal that those subject to higher years of compulsory schooling have lower smoking-related outcomes, suggesting that higher education significantly lowers tobacco consumption in LMICs. The effect is primarily observed for women, where, for instance, higher compulsory schooling reduces the probability of smoking by 23% and the number of cigarettes smoked by 27%.

**Conclusions:**

The study’s results establish the causal link between education and smoking behavior in LMICs. This significant impact suggests that education policy is still an important tool to help reduce tobacco consumption, especially in settings where the average level of education is not high initially. Moreover, discouraging men from smoking requires other measures to complement education policy.

**Implications:**

Education might help reduce tobacco consumption. However, studies—primarily for developed countries—find mixed results. This paper investigates the causal role of education on smoking in LMICs. Education reduces tobacco consumption, especially for women. Thus, education policy can be effective in low-education settings. Nonetheless, education policy should be accompanied by other policies to discourage men from smoking.

## Introduction

Smoking is associated with several socioeconomic factors, and its prevalence is unequally distributed across certain groups leading to inequalities in the consumption of tobacco products. One primary dimension is educational inequality—the difference between the smoking prevalence among individuals with the lowest and highest level of education—which has extensively been studied in the literature. For instance, it has been shown that educational inequalities varied to a large extent across low-and-middle-income countries (LMICs);^1^ the educational inequality occurs later in women than in men in European countries;^2^ the educational inequality exists only in certain age groups for immigrants coming from different countries of origin;^3^ the smoking cessation rate is higher among highly educated persons compared to those low educated,^4,5^ and in addition, higher education is associated with lower prevalence of smoking.^6^ However, the studies investigating educational inequality are associative as they typically compare smoking prevalence among individuals with different levels of education unconditionally.

Despite being useful in a descriptive sense, these studies do not establish a causal link between education and smoking, as they do not consider the potential endogeneity. That is, education might affect smoking behavior, at the same time smoking might also affect education, or both can be affected by another common factor. For instance, as income and education levels of individuals are highly correlated in general, comparing the outcomes of groups with different years of education may well be capturing the effects of varying income levels on smoking rather than education. However, establishing a causal link between education and smoking is essential when proposing policy recommendations.

Identifying causal effects in health economics is essential, as better health outcomes are considered an investment in human capital. Not smoking is an example of such an investment. Thus, quitting or at least reducing the amount of tobacco consumed leads to substantial human capital improvements, which further generate positive external effects on economic welfare, both through increased productivity of an individual and through a lower burden on the national health system.

The causal studies on the effect of education on tobacco consumption rely on different strategies to identify the causal impact. The main concern of the causal studies is to find an exogenous factor that would lead to an increase in education while not directly affecting tobacco consumption behavior. Then, after controlling for potential covariates, the difference between tobacco consumption outcomes of individuals exposed to an exogenous increase in education with those of similar characteristics but not subject to higher education are compared.

Several papers provide recent evaluations of studies aiming to measure education’s causal impact on health outcomes, including smoking.^7,8^ As shown in a review of the causal evidence from randomized controlled trials, twin studies, and quasi-experiments (the majority of which use the variation in the years of compulsory schooling), the evidence in favor of education lowering the probability of being a current smoker is mixed.^7^ For instance, the higher years of education did not affect the prevalence of smoking in Britain^9,10^; Germany^11,12^; Australia^13^; and Canada;^14^ while reducing the prevalence of smoking for men in Northern Ireland^10^; for women in Germany^15^; France^16^; and the United States.^17^ Meanwhile, a recent study for the United States also does not find a significant impact of higher education on the probability of ever smoking.^18^

As observed, the majority of the studies offer evidence from developed economies. A survey reviewing the studies that rely on the compulsory schooling policy to identify the causal effect of education on health outcomes—including smoking—reveals that out of 25 countries for which the evidence is reported, all but two are developed economies.^8^ Among the few LMICs, education has no causal effect on smoking in Turkey^19,20^; while it reduces smoking prevalence in Vietnam^21^ and China.^22^

In this study, we aim to fill the gap in the literature by estimating the causal impact of education on smoking for a set of LMICs. To this end, by using the data from the Multi-Indicator Cluster Surveys (MICS)—household-level surveys conducted in LMICs—sponsored by the UNICEF, and from the World Bank—on the years of compulsory schooling—, we identify 12 LMICs that have increased the years of compulsory schooling in the last two decades and have available household surveys. Then, we compare the smoking-related outcomes of 15–49-year-old individuals subject to higher years of compulsory schooling with the outcomes of those subject to lower years of compulsory schooling.

## Methods

### Data

In this paper, we use data from MICS surveys which are nationally representative and comparable across countries. These surveys provide a robust and valuable data source across the globe, having covered 116 countries.^23^ In addition to household-level indicators, the survey has separate questionnaires for women and men, and one section of the survey is devoted to the tobacco consumption behavior of the participants. While all rounds include a women’s module, not all surveys across MICS rounds and countries include a men’s module. Similarly, not all surveys have a module on tobacco consumption. Overall, we use data for 15–49-year-old individuals from 15 distinct surveys covering 12 LMICs—which have changed their compulsory schooling policy over the period covered by the surveys—with surveys including a tobacco use module. The list of countries, surveys, and number of observations are presented in Table 1.

**Table 1.** The Available Surveys and Number of Observations

		Female sample				Male sample		
Country and survey year/MICS round		4	5	6	Total	5	6	Total
Chad	2019			22,561	22,561		6931	6931
Costa Rica	2018			7502	7502			
Dominican Republic	2014		29 200		29 200			
El Salvador	2014		13 350		13 350			
Ghana	2017			14 374	14 374		5323	5323
Guinea Bissau	2014, 2019		10 234	10 945	21 179	4232	2805	7037
North Macedonia	2011	3831			3831			
Mongolia	2010, 2013, 2018	8762	12 830	10 794	32 386	5714	4477	10,191
Paraguay	2016		7311		7311			
Senegal	2015		9404		9404	3802		3802
Tonga	2019			2903	2903		1232	1232
Ukraine	2012	8006			8006			
Total		20,599	82 329	69 079	172 007	13 748	20 768	34 516

The sample includes countries that have changed compulsory schooling duration in the last two decades and have surveys available. Questionnaires for males are only available in some countries in the 5th and 6th survey rounds.

The information regarding the changes in the compulsory policy across countries and the affected cohorts are given in Table 2.

**Table 2.** Change in Compulsory Schooling Policy Across Countries

Country	Policy change in	Old/New policy (years)	Policy effective from (birth cohort ≥)
Chad	2006	6/10	1994
Costa Rica	2011	11/13	1994
Dominican Republic	2010	9/15	1995
El Salvador	2013	9/12	1997
Ghana	2007	9/11	1992
Guinea Bissau	2001	6/9	1989
North Macedonia	2010	9/13	1995
Mongolia	2006	9/12	1990
Paraguay	2011	9/13	1996
Senegal	2004	6/11	1992
Tonga	2012	6/8	2000
Ukraine	2003	10/11	1987

The policy change time is the year in which the years of compulsory schooling were extended. Old (new) policy refers to the years of compulsory schooling in the old (new) policy. The birth cohort from which the policy change is effective—ie, those individuals subject to higher compulsory schooling—is calculated by using the time of policy change, old policy, and the typical school start age. The typical school start age at the time of policy change is 7 for El Salvador and Mongolia, and 6 for the other countries. Source: World Bank.

The tobacco consumption module of the MICS includes a set of questions on smoking behavior. In this study, we use the following indicators: Ever smoked (binary 0/1); currently smokes (binary 0/1); number of cigarettes smoked in the last 24 hours; number of days smoked in last month; smoked a cigarette every day last month (binary 0/1); and consumed tobacco products other than cigarette in last month (binary 0/1).

### Estimation Strategy

In this study, we aim to investigate the causal effect of education on smoking behavior in LIMCs. To identify the causal effect, we use the changes in the compulsory schooling policy observed in LIMCs, forcing cohorts born after a particular year to have higher years of education than the previous cohorts, similar to.^24^ Focusing on a sample of observations from 15 to 49-year-old women and men across countries and identifying those who are subject to higher years of compulsory schooling—based on the year of birth—, we compare the smoking-related outcomes of individuals subject to higher years of compulsory schooling with those subject to lower years of compulsory schooling. To establish the causal link, we also control for potential covariates that could affect smoking behavior, such as income or wealth, age, marital status, area of residence, and region within the country. The effect of age is allowed to differ across countries, and fixed effects for age groups are also included to capture the smoking behavior that might be specific to a certain age group. The specifications also control for country by MICS round fixed effects to capture the factors common to all individuals in a country at the time of the survey, such as tax policy, cigarette prices, etc.

The identifying assumption is that after controlling for a wide range of potential covariates and introducing country and survey round specific fixed effects, the difference between the smoking-related outcomes of 15–49-year-olds subject to higher years of compulsory schooling and those of 15–49-year-olds subject to lower years of compulsory schooling gives the causal impact of having more education on the smoking behavior. In this respect, we estimate the following specification:


$$\begin{array}{l} S_{i,c,t}=\text{α}+\beta HCS_{i,c,t}+X_{i,c,t}^{\prime }\theta +\mu_{t}+\sigma_{c}+\gamma_{ag}+\rho_{r}+\varepsilon_{i,c,t} \end{array}$$ (1)

where *S*_*i*,*c*,*t*_ is a smoking-related outcome for individual *i*, from country *c*, and surveyed in round *t*. For binary smoking-related indicators of Ever smoked, Current smoker, Smoked every day last month, and Consumed tobacco products other than cigarette in last month, *S*_*i*,*c*,*t*_ takes the value of 1 if the individual belongs to that category and 0 otherwise. For the other smoking-related variables of number of cigarettes smoked in the last 24 hours, and number of days smoked in last month, *S*_*i*,*c*,*t*_ is a continuous variable taking non-negative values. *HCS*_*i*,*c*,*t*_ (higher years of compulsory schooling) indicates if the individual is affected by the policy change, taking the value of 1(0) for those subject to new (old) compulsory schooling policy. $X_{i,c,t}^{\prime }$ is a vector of individual-specific factors including age, wealth score, marital status (1 if married, 0 otherwise), whether one has given birth (1 if yes, 0 if no) (for females only), lives in a rural area (1 if rural, 0 if urban). The wealth score is calculated as a weighted index based on the assets of the households such as the ownership of consumer goods, dwelling characteristics, or other characteristics that are related to the household’s wealth, and it can be used to compare the relative ranking of the household in the wealth distribution in a given country and a survey round. *µ*_*t*_, *σ*_*c*_, *ρ*_*r*_, and *γ*_*ag*_ correspond to MICS round, country, region within the country, and age-group fixed effects, respectively. The age groups are defined as 15–24; 25–34; 35–44; and 45–49. The effect of age is also allowed to differ across countries. Also, the specification includes country by MICS round fixed effects, which control for factors common to all the individuals in a given country at the time of the survey, including any other policy that could affect smoking behavior. When presenting the results, these control variables are added to the model in a sequential way in three different columns. In the first column, the household wealth score and the country by survey round fixed effects are included as the control variables. In the second column, in addition to the first column, controls for age (allowing for differences across countries), marital status, and ever-given birth status (for women) are added. In the third—and the most comprehensive—column, in addition to first two columns, the region within the country, the area of residence (rural or urban), and age group fixed effects are included as control variables.

Estimations are carried out for women and men separately to account for the heterogeneous effects by gender, as reported by previous studies.^10,15,16^ In the baseline results, this model is estimated as a Linear Probability Model for binary smoking variables in order for the results to be comparable with the previous literature on the causal impact of education on smoking,^10–18^ where the standard errors robust to serial correlation and clustered at country-survey year level are used. Nonetheless, for a robustness exercise, the results from the Logit model for binary variables are also presented in Supplementary Appendix. For continuous variables, the baseline results correspond to the estimation of the linear model. The *β* coefficient in specification (1) gives the causal impact of education (induced by being subject to higher years of compulsory schooling) on smoking behavior for a set of binary and continuous smoking-related outcomes. We also calculate the impact size, which is the percent reduction in the prevalence of smoking-related indicators for those with higher education (subject to higher years of compulsory schooling) compared to the control group of those with lower education (subject to lower years of compulsory schooling) as the ratio of the estimated *β* coefficient to the corresponding sample proportion or sample mean.

## Results

First, we report the descriptive statistics of the main variables in the regression analyses for women and men in Table 3. The descriptive statistics reveal that 16.2% of women and 39.9% of the men in the sample ever smoked. Meanwhile, 3.6% (23.2%) of the women (men) are current smokers in the sample. Among the current smokers, on average, women smoke 6.7 cigarettes per day, while men smoke 10.0 cigarettes per day.

**Table 3.** Descriptive Statistics

	Women					Men				
*A. Binary* variables	Obs.	Sample proportion	Std. error	95% Confidence Interval		Obs.	Sample proportion	Std. error	95% Confidence Interval	
Ever smoked	172 007	16.20%	0.09%	16.03%	16.38%	34 516	39.89%	0.26%	39.37%	40.40%
Current smoker	172 007	3.62%	0.05%	3.53%	3.71%	34 516	23.21%	0.23%	22.76%	23.65%
Every-day smoker	172 007	2.44%	0.04%	2.37%	2.51%	34 516	19.21%	0.21%	18.80%	19.63%
Other tobacco products	172 007	0.81%	0.02%	0.77%	0.85%	34 516	5.54%	0.12%	5.30%	5.78%
HCS	171 527	29.70%	0.11%	29.49%	29.92%	34 407	40.84%	0.27%	40.32%	41.36%
Married	172 007	61.88%	0.12%	61.65%	62.11%	34 516	50.16%	0.27%	49.63%	50.68%
Gave birth	168 176	73.91%	0.11%	73.70%	74.12%					
Lives in rural area	172 007	45.96%	0.12%	45.72%	46.19%	34 516	53.13%	0.27%	52.60%	53.65%

	Women					Men				
*B. Continuous variables*	Obs.	Mean	Std. dev.	Min.	Max.	Obs.	Mean	Std. dev.	Min.	Max.
Number of cigarettes	171 994	0.241	1.774	0	64	34 490	2.318	5.48	0	60
* (among current smokers)*	*6209*	*6.666*	*6.661*	*0*	*64*	*7 985*	*10.011*	*7.259*	*0*	*60*
Days smoked last month	171 992	0.809	4.688	0	30	34 511	6.084	11.785	0	30
* (among current smokers)*	*6207*	*22.409*	*11.175*	*0*	*30*	*8006*	*26.229*	*8.390*	*0*	*30*
Age	172 007	29.688	9.592	15	49	34 516	29.359	10.089	15	49
Wealth score	172 007	0.089	0.99	−7.8	6.2	34 516	0.058	1.038	−3.2	6.1

The sample includes observations from 15 to 49-year-old women from all countries; and from 15 to 49-year-old men from Chad, Ghana, Guinea Bissau, Mongolia, Senegal, and Tonga. HCS stands for higher compulsory schooling and takes the value of 1 if the individual is subject to higher compulsory schooling policy. “Every-day smoker”: Smoked every day last month; “Other tobacco products”: Consumed other tobacco products last month; “Number of cigarettes”: Number of cigarettes smoked in last 24 hours. Confidence intervals for the population proportion are reported for binary variables.

Next, as a prior check of the validity of our approach, we show that being subject to more years of compulsory schooling leads to a rise in the amount of education received, as provided in Supplementary Appendix. Establishing the impact of compulsory schooling on education, we next turn to our main specification to test the causal impact of education on smoking using the exogenous variation in education triggered by the increase in compulsory schooling. The estimation results are presented in Table 4 for women and men separately. Each cell reports the estimated policy effect—that of education on smoking behavior—which is the *β* coefficient in specification (1) estimated for models with different control variables (in columns) and for the smoking-related indicator (in rows). As described in the empirical strategy, the third column shows the results from the most comprehensive specification with the highest number of control variables, and the findings are discussed based on this column. Also, the size of the estimated policy impact is calculated for the third column, and for those indicators with a significant *β* coefficient. The panel A in the table reports the results of Linear Probability Model estimated for binary smoking-related indicators. For a robustness checks, Logit model results are also provided in Supplementary Appendix for binary indicators. Meanwhile, Panel B reports the estimation results for continuous variables.

**Table 4.** Estimation Results—The Impact of Education on Smoking

Sample:	Women			Men		
	(1)	(2)	(3)	(1)	(2)	(3)
*A: Binary indicators of smoking behavior—Result from Linear Probability Model estimations*						
	Ever smoked			Ever smoked		
HCS Coefficient	−0.0361***	−0.0418***	−0.0276***	−0.192***	−0.0973***	−0.0324*
Standard error	(0.00683)	(0.0101)	(0.00998)	(0.0147)	(0.0180)	(0.0193)
*Impact size*			*−18%*			*−8%*
	Current smoker			Current smoker		
HCS Coefficient	−0.0160***	−0.0111***	−0.00772***	−0.153***	−0.0852***	−0.0220
Standard error	(0.00205)	(0.00255)	(0.00288)	(0.0152)	(0.0195)	(0.0238)
*Impact size*			*−23%*			
	Smoked every day in last month			Smoked every day in last month		
HCS Coefficient	−0.0130***	−0.00546***	−0.00378**	−0.132***	−0.0718***	−0.0163
Standard error	(0.00151)	(0.00176)	(0.00187)	(0.0146)	(0.0180)	(0.0232)
*Impact size*			*−16%*			
	Other tobacco products last month			Other tobacco products last month		
HCS Coefficient	0.000613	−0.00105	−0.00308*	−0.0380***	−0.0172***	−0.0140**
Standard error	(0.00128)	(0.00121)	(0.00175)	(0.00482)	(0.00517)	(0.00704)
*Impact size*			*−35%*			*−25%*
*B: Continuous indicators of smoking behavior—Result from OLS estimations*						
	Number of cigarettes in last 24 hours			Number of cigarettes in last 24 hours		
HCS Coefficient	−0.129***	−0.0580***	−0.0459***	−1.872***	−0.861***	−0.251
Standard error	(0.0162)	(0.0162)	(0.0172)	(0.209)	(0.202)	(0.240)
*Impact size*			*−27%*			
	Number of days smoked in last month			Number of days smoked in last month		
HCS Coefficient	−0.408***	−0.199***	−0.139**	−4.144***	−2.274***	−0.564
Standard error	(0.0482)	(0.0567)	(0.0604)	(0.442)	(0.553)	(0.702)
*Impact size*			*−22%*			
Obs.	171 532	167 701	158 939	34 408	34 408	34 408

Each cell reports the results of a separate regression where a smoking-related indicator (top line) is regressed on the Higher Compulsory Schooling (HCS) indicator and a list of control variables in each column. The coefficient of the HCS −*β*—in Specification 1—is the policy effect of being subject to higher years of compulsory schooling on smoking behavior. Column (1): controls for wealth score and country by MICS round fixed effects. Column (2): (1) + controls for age (allowing for differences across countries), whether married, and whether has given birth (for women). Column (3): (2) + controls for the region within the country, lives in a rural area, age group fixed effects. Standard errors clustered at birth year by country level are given in parentheses. ***, **, and * refer to statistically significant coefficients at 1, 5, and 10%, respectively. The impact size, which is the percent reduction in the prevalence of smoking-related indicators for those with higher education compared to the control group of those subject to lower education, is calculated as the ratio of the estimated coefficient to the sample proportion (sample mean) in Panel A (B) for the corresponding sample used in the estimation. The policy impact is reported only for Column 3 and for cases where the coefficient of HCS is statistically significant.

Focusing on women, those subject to higher years of compulsory schooling have significantly lower smoking-related outcomes, indicating the causal role of education in reducing the prevalence of smoking. Women with higher education are less likely to have ever smoked cigarettes, currently smoke, and consume other tobacco products. Also, they smoke fewer cigarettes and smoke for fewer days in a month. The impact size of the policy is also sizeable. Accordingly, the policy reduces the probability of having ever smoked by 18%, the probability of currently smoking by 23%, the number of cigarettes smoked in the last 24 hours by 27%, and the probability of consuming other tobacco products by 35% among women.

The effect of education on smoking behavior is less pronounced for men. The policy reduces the probability of ever having smoked by 8% in the Linear Probability Model, but this finding is not robust as no significant effect is reported in the Logit estimations provided in Supplementary Appendix. The policy, on the other hand, reduces the probability of consuming other tobacco products by 25%. Meanwhile, the policy does not significantly affect the likelihood of being a current smoker, number of cigarettes smoked, or the number of days smoked last month. Note that the significant effect of education on several smoking outcomes observed in columns 1 and 2 disappear in column 3, where potential cofounders of the region of residence within the country, area of residence (rural vs. urban), and age group fixed effects are controlled.

## Discussion

The study’s findings reveal that education causally reduces smoking prevalence in LMICs. From this perspective, the study contributes to the scarce literature on the impact of education on smoking in the setting of LMICs. Meanwhile, the effects are heterogenous both regarding the smoking outcome considered and by gender. First, the effect of education is more substantial on current smoking behavior—being a current smoker and the number of cigarettes smoked—and less on the probability of ever smoking. Second, in the sample of countries, the policy effect is much stronger for women than men. Note that as the MICS surveys are mainly developed for women and children, men are only sampled in a few numbers of countries and only in a few survey rounds. Moreover, when they are sampled, the sample size of men is much lower than women, at times as low as one-third of the number of women sampled in the same survey round of a country. Therefore, this result may partly be driven by a lower representation of men. On the other hand, as Supplementary Table A.1 in Supplementary Appendix shows, compulsory schooling leads to a higher increase in education variables for women, which may help explain the heterogeneity between women and men. There could also be another source of heterogeneity stemming from the level of initial compulsory schooling policy, that is, the size of the policy effect of higher compulsory schooling could be different whether the new policy extends it beyond primary school or beyond middle school, for instance. However, in our empirical strategy, the use of country-fixed effects, which control for country-specific factors including the initial level of compulsory schooling, does not allow us to analyze this sort of policy heterogeneity in this setting.

These findings point to several policy implications. First, while the studies for advanced countries provide mixed results on the causal impact of education on smoking, as discussed in the introduction, our significant results suggest that increased years of education can still be an important factor in reducing smoking prevalence, especially in the settings where the average years of education of the population are lower than that of advanced countries. Second, the results suggest that education should also be accompanied by other measures to motivate the men in these countries to reduce their consumption or to quit.

## Conclusion

Educational inequalities are often cited as essential drivers of smoking behavior, suggesting that increasing the education level or the years of schooling may help reduce tobacco consumption. However, the causal investigations establishing the link from education to smoking outcomes are scarce in the context of lower-income countries. In this study, by exploiting the exogenous increase in education caused by the rise in the duration of compulsory schooling, we investigate the causal role of education on smoking behavior for a panel of 12 LMICs. By using detailed micro-level household surveys, we find that an exogenous increase in education significantly reduces smoking-related outcomes, primarily for women. Meanwhile, the limited effect of education reported for men should be considered cautiously as the sample size of men are much more limited compared to the women in these surveys. Overall, the findings imply that investing in education can still be an effective tool to fight tobacco consumption, especially in settings where the average level of education is not high enough.

## Supplementary Material

A Contributorship Form detailing each author’s specific involvement with this content, as well as any supplementary data, are available online at https://academic.oup.com/ntr.

ntad056_suppl_Supplementary_Appendix

## Data Availability

The data is available upon request.

## Appendix

The appendix is available as a supplementary file.
