# Supplementary material for: Causal Effect of Education on Tobacco Use in Low-and-Middle-Income Countries
Source: Nicotine Tob Res. 2023 Apr 5;25(8):1474–80. doi: 10.1093/ntr/ntad056 (PMC10632550; doi:10.1093/ntr/ntad056)
Supplement: ntad056_suppl_Supplementary_Appendix [file ntad056_suppl_supplementary_appendix.docx]

Supplementary File for

*Causal Effect of Education on Tobacco Use*

*in Low-and-Middle-Income Countries*

Appendix

1. *The link between higher years of compulsory schooling and education*

We test whether being subject to more years of compulsory schooling leads to a rise in the amount of education received. Given that the surveys do not explicitly report the years of schooling, we use two variables to measure the amount of education received. The first variable is “Above Primary School” and takes the value of 1 if the highest level of education received is above primary school and 0 otherwise. The second variable is the “Level of Education” where the values 1, 2, and 3 represent Primary School, Secondary School, and Above Secondary School, respectively. The results provided in Table A.1 show that women and men subject to higher years of compulsory schooling have statistically higher education outcomes. For instance, the policy increases the probability of women having a degree above the primary school by 9.9% and the mean level of education by 0.12. Meanwhile, the size of the impact are lower for the men in the sample.

1. *Robustness Check: Logit model for binary smoking indicators*

In this robustness check, we use the Logit model to test the impact of education on binary smoking related variables. On the contrary to the Linear Probability Model (the baseline estimation), given that Logit model is a nonlinear model, the estimated coefficients does not directly give the size of the policy effect. To estimate the policy effect, we first calculate the estimated probability of a smoking related indicator (its proportion) for those who are subject to higher years of compulsory schooling and for those who are not subject to higher years of compulsory schooling. Then, the impact size is calculated as the percentage difference between the smoking outcome probabilities estimated for these two groups. The results presented in Table A.2 reveal that the significance of the policy effect is similar to the baseline estimations. The only difference is that the Logit estimates point to a higher policy effect for being a current smoker and for being an everyday smoker amongst the women. Also, the significant effect found for the probability of ever smoked for men in the baseline estimations is not significant in the case of Logit model.

| Table A.1: The impact of higher compulsory schooling on education | | | | |
| --- | --- | --- | --- | --- |
| Sample: | Women | | Men | |
|  | Above Primary School | Level of Education | Above Primary School | Level of Education |
| HCS | 0.0986*** | 0.123*** | 0.0399** | 0.0609*** |
|  | (0.0116) | (0.0207) | (0.0177) | (0.0179) |
| Obs. | 153,252 | 136,740 | 14,510 | 12,837 |
| Note: Each cell reports the results of a separate regression where either Above Primary School (1 if above primary school, 0 otherwise) or Level of Education (1: Primary, 2: Secondary, 3: Above Secondary) (top line) is regressed on the Higher Compulsory Schooling (HCS) indicator and a list of control variables. The control variables include the country by survey round fixed effects, region of country fixed effects, area of residence (urban vs. rural), and wealth score. The sample includes 15-49 age group in "Above Primary School" columns and 18-49 age group in "Level of Education" columns. Standard errors clustered at the birth year by country level are given in parenthesis. ***, **, and * refer to statistically significant coefficients at 1, 5, and 10%, respectively. | | | | |

| Table A.2: Estimation results - The impact of education on smoking | | | | | | |  |  |
| --- | --- | --- | --- | --- | --- | --- | --- | --- |
| Binary indicators of smoking behavior – Result from Logit estimations | | | | | | | |  |
| Sample: | Women | | | Men | | | |  |
|  | (1) | (2) | (3) | (1) | (2) | (3) | |  |
|  | Ever smoked | | | Ever smoked | | | |  |
| HCS Coefficient | -0.398*** | -0.437*** | -0.295*** | -1.296*** | -0.621*** | -0.112 | |  |
| Standard error | (0.0816) | (0.0994) | (0.104) | (0.0926) | (0.104) | (0.103) | |  |
| *Impact size* |  |  | *-18%* |  |  |  | |  |
|  | Current smoker | | | Current smoker | | | |  |
| HCS Coefficient | -0.719*** | -0.622*** | -0.448*** | -1.251*** | -0.731*** | -0.163 | |  |
| Standard error | (0.107) | (0.115) | (0.136) | (0.138) | (0.163) | (0.163) | |  |
| *Impact size* |  |  | *-33%* |  |  |  | |  |
|  | Smoked every day in last month | | | Smoked every day in last month | | | |  |
| HCS Coefficient | -0.954*** | -0.684*** | -0.452*** | -1.264*** | -0.725*** | -0.155 | |  |
| Standard error | (0.134) | (0.128) | (0.144) | (0.157) | (0.181) | (0.183) | |  |
| *Impact size* |  |  | *-34%* |  |  |  | |  |
|  | Other tobacco products last month | | | Other tobacco products last month | | | |  |
| HCS Coefficient | 0.0932 | -0.334** | -0.362** | -1.248*** | -0.766*** | -0.462*** | |  |
| Standard error | (0.178) | (0.132) | (0.143) | (0.140) | (0.153) | (0.148) | |  |
| *Impact size* |  |  | *-29%* |  |  | *-28%* | |  |
| Note: Each cell reports the results of a separate logit model estimation where a smoking-related indicator (top line) is associated with the Higher Compulsory Schooling (HCS) indicator and a list of control variables in each column. Column (1): controls for wealth score and country by MICS round fixed effects. Column (2): (1) + controls for age (allowing for differences across countries), whether married, and whether has given birth (for women). Column (3): (2) + controls for the region within the country, lives in a rural area, age group fixed effects. The impact size is calculated as the percentage difference between the smoking outcome probabilities estimated for the group not affected by the policy and for the group affected by the policy change (those subject to higher years of compulsory schooling). The policy impact is reported for Column 3 (most comprehensive specification) and only for cases where the coefficient of HCS is statistically significant. Standard errors clustered at birth year by country level are given in parenthesis. ***, **, and * refer to statistically significant coefficients at 1, 5, and 10%, respectively. | | | | | | | |  |
|  |  |  |  |  |  |  |  |  |
|  |  |  |  |  |  |  |  |  |
